# Validation of the Somnolyzer 24×7 automatic scoring system in children with suspected obstructive sleep apnea

**DOI:** 10.3389/fmed.2025.1617530

**Published:** 2025-06-18

**Authors:** Ignacio Boira, Violeta Esteban, José Norberto Sancho-Chust, Esther Pastor, Paula Fernández-Martínez, Anastasiya Torba, Eusebi Chiner

**Affiliations:** Department of Pneumology, Sleep Unity, San Juan de Alicante University Hospital, Alicante, Spain

**Keywords:** obstructive sleep apnea, sleep disorders, children, Somnolyzer, polysomnography, artificial intelligence

## Abstract

**Introduction:**

Manual scoring of polysomnography data is a laborious and complex process. Automatic scoring by current computer algorithms shows high agreement with manual scoring. The primary objective of this study was to measure the overall validity of the Somnolyzer 24×7 automatic polysomnography scoring system in children.

**Materials and methods:**

We conducted a single-center, prospective, observational study in children undergoing diagnostic polysomnography for suspected obstructive sleep apnea (OSA) from December 2023 to December 2024. We included children aged three to 15 years with suspected obstructive sleep apnea (OSA). Each polysomnogram was scored manually by three experts and automatically by the Somnolyzer 24×7 system.

**Results:**

Our analysis included 75 children (60% girls), of whom 9% did not have OSA, 20% had mild OSA, 31% moderate OSA, and 40% severe OSA. There was a high level of agreement between manual and automatic scoring of the respiratory disturbance index (RDI). The mean correlation (Pearson correlation coefficient) of RDI scored by the three experts was 0.93 (95% confidence interval [CI] 0.92–0.95), similar to the correlation between manual and automatic scoring (0.92, 95% CI 0.90–0.94). The correlation between the different manual scorings and between manual and automatic scoring was maintained in the different sleep stages (N1: 0.93 vs. 0.90, N2: 0.76 vs. 0.73, N3: 0.72 vs. 0.76, REM: 0.86 vs. 0.82).

**Conclusion:**

The Somnolyzer 24×7 automatic scoring system shows strong correlation with manual scoring in respiratory events and sleep architecture. Our results suggest this system could be used for polysomnography scoring in children.

## 1 Introduction

Obstructive sleep apnea (OSA) is characterized by partial or total obstruction of the upper airway, which alters alveolar ventilation and sleep quality (1–3). In children, OSA has an estimated prevalence of 1–4%. The most common etiology is adenotonsillar hypertrophy (4), which defines the predominant phenotype. The second main phenotype involves concomitant diseases or comorbidities (with or without tonsillar hypertrophy) such as craniofacial abnormalities, neuromuscular diseases, or complex syndromes (Down syndrome, Prader-Willi syndrome, or Beckwith-Wiedemann syndrome) (5). In addition to anatomical characteristics, childhood obesity is a growing risk factor for sleep disorders in the pediatric population (6). Pediatric OSA has been associated with the development of neurocognitive and behavioral disorders, delayed growth, cardiovascular diseases, metabolic disorders, and reduced quality of life due to a proinflammatory state, increased sympathetic activity, altered coagulation, and oxidative stress (7, 8). For these reasons, early diagnosis is crucial. While polysomnography is the diagnostic gold standard (9), home respiratory polygraphy has demonstrated similar validity in detecting sleep-disordered breathing in children (10).

Manual scoring of polysomnographic data is a laborious and complex process with marked interscorer variability, especially in the identification of arousals and sleep stages (11). In response to these limitations, computerized algorithms were developed to analyze electrophysiological signals and classify sleep stages and respiratory events, achieving a high level of agreement with manual scoring by sleep medicine experts. Notably, computerized algorithms have been validated with manual scoring as a reference standard (12). An automated scoring system could be considered a valid alternative if the level of agreement between automated and manual scoring were comparable to the level of interscorer agreement observed with manual scoring. Recently, sleep medicine has benefited from Artificial Intelligence (AI), which offers innovative solutions to the limitations of traditional diagnostic methods. AI has enabled the classification of sleep stages and the detection of sleep-disordered breathing through the analysis of complex physiological data. AI can process datasets, identify patters and make predictions with high accuracy respect to manual scoring. One of the limitations of AI in pediatric sleep is the lack of pediatric dataset for training AI model. This can limit the generalizability of AI models to childen (13, 14).

The following machine learning algorithms have been employed in pediatric sleep apnea: Convolutional Neural Networks (CNNs), Support Vector Machines (SVM), Random Forest (RF), Transformer-based Model long short-term memory (LSTM)-based and Sleep Staging Model (CSleep Net). These algorithms have achieved high levels of accuracy (13–15). Moeller and colleagues (16, 17) reported that U-Sleep performed an overall accuracy in sleep stages of 83.9% and a kappa value of 0.77 comparable to human experts. Somaskandhan and colleagues (16, 18) developed a combined convolutional and long short-term memory neural network architecture. Their model achieved an overall accuracy of 84.1% (Cohen’s kappa *κ* = 0.78), comparable to interrater reliability between manual scorers without evidence of difference between children with sleep-disordered breathing and control groups.

The most widely employed computer-assisted sleep staging system is the Somnolyzer 24×7, which has Food and Drug Administration (FDA) approval and follows the American Academy of Sleep Medicine (AASM) scoring guidelines (19). This supervised system analyzes sleep stages, respiratory events, desaturations, limb movement, and arousals, but cannot evaluate paroxysmal activity or bruxism. The steps of the analysis are: (1) artifact processing (minimization, identification, channel selection), (2) feature extraction (slow wave, sleep spindles, k complexes, delta, theta, alpha, slow and fast beta background activities, dominant alpha frequency, arousals, various artifact types, slow and rapid eye movements, eye blinks, tonic and transient muscle activity), (3) AI classifier (bi-directional long-short-term memory recurrent neural network [RNN]), and (4) rule-based sub-classification of non-rapid eye movement (NREM) sleep (configuration option). Based on RNN probability, the system uses traffic light color coding to indicate the confidence in its scoring (green: high confidence, yellow: medium confidence, red: low confidence). This automatic scoring system has been validated in several adult studies (20–28), achieving very high agreement with manual analysis (Table 1). However, no studies have validated the Somnolyzer system in children.

**Table 1:** Summary of the results of the most relevant articles comparing manual scoring with Somnolyzer system scoring.

Anderer et al. (21)	Epoch-by-epoch agreement: 80% (Cohen’s kappa: 0.72) between Somnolyzer 24×7 and human expert scoring. Inter-rater reliability (2 experts): 77% (Cohen’s kappa: 0.68), Inter-rater reliability (2 Somnolyzer 24×7 analysis with quality control by 2 human experts): close to 1 (Cohen’s kappa: 0.991).
Barbanoj et al. (22)	Epoch-by-epoch agreement: 80%.
Anderer et al. (23)	Epoch-by-epoch agreement between manual scoring 1 and semi-automated scoring 1: 82% (kappa: 0.76) and between manual scoring 2 and semi-automated scoring 2: 81% (kappa 0.75). Cohen’s kappa between automated and manual scoring 1: 0.71. Cohen’s kappa between automated and manual scoring 2: 0.72. Spearman rank correlation between manual and semi-automated scoring: N1(%): 0.76, N2(%): 0.74, N3(%): 0.89, REM(%): 0.85.
Griessenberger et al. (24)	Overall agreement of all epochs: 80.9% (Cohen’s kappa: 0.69). Significant correlation in light sleep (*r* = 0. 480) and deep sleep (*r* = 695).
Punjabi et al. (20)	Pearson correlation coefficient (*r*) of AHI between manual and automated score: 0.93 (95% CI 0.91–0.96). Average bias in AHI: 2.48 events/h (95% CI 0.40–4.55). Pearson correlation coefficient between manual and automated score of sleep architecture (N1: 0.63, 95% CI 0.57–0.70; N2: 0.66, 95% CI 0.59–0.74; N3: 0.65, 95% CI 0.57–0.7; REM: 0.92, 95% CI 0.91–0.94).
Magnusdottir et al. (25)	Sensitivity: 93%, specificity: 79%, Cohen’s kappa: 0.74, agreement: 87%
Bakker et al. (26)	Intraclass correlation coefficient for all sleep stages between automatic and manual score: 0.91 (Wake: ≥ 0.93, N1: 0.72–0.74, N2: 0.88–0.89, N3: 0.85–0.94, REM: 0.96–0.97).
Cheng et al. (27)	Accuracy of sleep staging: 77% (76.8–77.35), Cohen’s kappa: 0.68, accuracy: 72.57%, recall: 76.09%. Correlation coefficient of wake: 0.91, N1: 0.65, N2: 0.86, N3: 0.73, REM: 0.85.
Gomes et al. (28)	Pearson correlation coefficient (r) of AHI: 0.98, OAI: 0.87, CAI: 0.88, ODI: 1. AUC for altered OSA: 0.85, mild OSA: 0.70, moderate OSA: 0.73 and severe OSA: 0.93.

AHI, apnea-hypopnea index; N(1,2,3), non-rapid eye movement stages of sleep; OAI, obstructive apnea index; ODI, oxygen desaturation index; OSA, obstructive sleep apnea; REM, rapid eye movement stage of sleep.

The main objective of this study was to determine the overall validity of the Somnolyzer 24×7 automatic system for reading pediatric polysomnography data. Secondary objectives included evaluating the validity of the Somnolyzer system for classifying sleep stages and identifying respiratory events.

## 2 Materials and methods

### 2.1 Study design

We conducted a single-center, prospective, observational study in children undergoing diagnostic polysomnography for suspected OSA from December 2023 to December 2024.

### 2.2 Study population

Inclusion criteria were age 3–15 years, polysomnography due to suspected OSA, and high-quality recording (at least 6.5 h, with at least 3 h of sleep). Children with a primary diagnosis other than OSA (insomnia, circadian rhythm disturbances, parasomnias, narcolepsy, idiopathic or recurrent hypersomnia, restless legs syndrome, and periodic leg movement disorder) were excluded from the study. Split-night recordings were also excluded.

### 2.3 Variables

We collected demographic variables, clinical variables, and all sleep study parameters (respiratory disturbance index [RDI]; hypopnea index [HI]; obstructive apnea index [OAI]; central apnea index [CAI]; oxygen desaturation index [ODI]; arousal index; percentage of total sleep time with oxygen saturation below 90% [T90]; total sleep time; sleep efficiency; and time in N1, N2, N3, and REM stages). A respiratory event is scored as an apnea if there is a drop in peak signal excursion by ≥90% of the pre-event baseline and the duration of this drop lasts at least the minimum duration specified for obstructive, mixed, or central apnea. A central apnea is further defined as an event that is associated with absent inspiratory effort throughout the event, and at least one of the following: the event lasts 20 s or longer or the event lasts at least the duration of two breaths during baseline breathing and is associated with an arousal or ≥3% oxygen desaturation. An obstructive apnea is defined as a complete cessation of airflow due to upper airway obstruction, accompanied by continued respiratory effort, lasting at least 10 s or the duration of two baseline breaths, and also associated with an arousal or a decrease in oxygen saturation of 3% or more. A respiratory event is scored as a hypopnea if the peak signal excursions drop by ≥30% of the pre-event baseline, the duration of this drop lasts for at least two breaths and there is ≥3% desaturation from pre-event baseline or the event is associated with an arousal (19). We classified OSA severity according to AHI (mild OSA: AHI of 1–5/h^−1^, moderate OSA: AHI of 5–10 h^−1^, severe OSA: AHI above 10/h^−1^).

All polysomnograms were performed using the SomnoStar Alfa (SensorMedics, CA, USA). For automatic scoring, we used the Somnolyzer system (Philips-Respironics, Murrysville, PA, USA, version 4.0). Three pulmonologists specialized in sleep medicine performed independent manual readings following the AASM criteria for scoring sleep stages and respiratory events (19). All the scorers were blinded to each other’s assessment and to the AI output. No inter-rater variability controls were performed.

### 2.4 Sample size calculation

The sample size was adjusted to 75 children, given a confidence level (1-*α*) of 95%, a precision (d) of 3% and a power of 80%.

### 2.5 Statistical analysis

We analyzed the concordance between manual scoring and automatic scoring. After applying the Kolmogorov–Smirnov test for normality of distribution, we used parametric (Student’s t) or non-parametric (McWhitney) tests to compare continuous variables. We used the Chi-square test or Fisher’s test for categorical variables. To evaluate differences between manual and automatic scoring, we applied analysis of variance (ANOVA). To evaluate the level of correlation between manual and automatic scoring, we used either the Pearson or Spearman correlation coefficient, depending on the distribution of the variable. We used intraclass correlation coefficients (ICCs) and the Bland–Altman method to evaluate agreement (29). For all comparisons, *p* values below 0.05 indicated statistical significance. All statistical analyses were performed using IBM SPSS Statistics v25 (Arming, NY).

### 2.6 Ethical considerations

The study was conducted in accordance with the Declaration of Helsinki and approved by the Clinical Research Ethics Committee of San Juan de Alicante University Hospital (ref 23/016). Participants’ parents or legal guardians signed an informed consent form.

## 3 Results

Our study included 75 children (60% girls) with a mean age of 8 (standard deviation [SD] 4) years and a mean body mass index (BMI) of 17.5 (SD 3.8) kg/m2. Seven children (9%) were not diagnosed with OSA, 15 (20%) had mild OSA, 23 (31%) had moderate OSA, and 30 (40%) had severe OSA. Normally distributed variables were BMI; RDI; number of hypopneas; arousal index; percentage of sleep in N1, N2, and N3; duration of N1, N2, and N3; and total sleep time. Table 2 shows the polysomnographic parameters of manual and automatic scoring and the comparison of mean scores (ANOVA) between the scoring methods. Regarding to respiratory events, there were no significant differences in the means of OAI, CAI and HI between manual and automatic corrections and these differences were not relevant in the RDI (*p* = 0.98). Concerning to sleep stages, the N2 phase evidenced the greatest difference in mean scores compared to the other sleep stages, although this difference did not reach statistical significance. Moreover, there were also no significant differences in the comparison of means for ODI, arousals index, T90, total sleep time, and there was a trend toward statistical significance in sleep efficiency.

**Table 2:** Analysis of variance of manual and automatic scoring.

	Expert 1	Expert 2	Expert 3	Somnolyzer	*p* value
RDI, events/h (mean ± SD)	9.5 ± 7.2	9.7 ± 6.7	9.6 ± 6.9	9.9 ± 6.4	0.98
Total obstructive apneas (median [IQR])	11 (2–22)	9 (3–21)	16 (5–27)	13 (7–25)	0.48
OAI, events/h (median [IQR])	1.7 (0.4–3.2)	1.3 (0.3–2.8)	2.7 (1.5–6.1)	2.2 (1–4)	0.30
Total central apneas (median [IQR])	1.5 (0–4)	1 (0–2)	2 (0.5–3)	2 (0.5–6)	0.37
CAI, events/h (median [IQR])	0.2 (0–0.5)	0.1 (0–0.2)	0.2 (0–0.6)	0.3 (0.1–0.8)	0.40
Total hypopneas (mean ± SD)	45 ± 32	50 ± 45	42 ± 29	40 ± 26	0.26
HI, events/h (median [IQR])	5.8 (2.6–9.9)	6.2 (1.9–10.6)	4.8 (1.7–9.8)	5.3 (2.9–8.4)	0.28
N1, minutes (median [IQR])	60 (18–100)	71 (15–120)	54 (12–97)	46 (27–99)	0.65
N1, % (mean ± SD)	15.8 ± 12.2	16.2 ± 9.5	14.8 ± 9.7	15.5 ± 13.3	0.88
N2, minutes (mean ± SD)	204 ± 66	175 ± 48	163 ± 42	198 ± 58	0.16
N2, % (mean ± SD)	50 ± 14	44.2 ± 10.8	43.6 ± 10.8	48 ± 15	0.34
N3, minutes (mean ± SD)	98 ± 60	102 ± 78	109 ± 87	103 ± 81	0.65
N3, % (mean ± SD)	23.1 ± 13.3	24.2 ± 12.1	25.8 ± 10.6	24 ± 17	0.71
REM, minutes (median [IQR])	36.5 (20–70)	42 (19–62)	45 (17–59)	38 (15–66)	0.88
REM, % (median [IQR])	8.5 (5–17.2)	10 (5–15)	11 (6–15)	9 (3–16)	0.81
ODI, events/h (median [IQR])	5.1 (1.7–11.9)	5.6 (2–13.3)	6 (2.7–12.8)	6.4 (2.3–14)	0.62
Arousal index, events/h (mean ± SD)	8.6 ± 5.3	10.6 ± 8.2	11.1 ± 8.2	9.6 ± 5.1	0.68
T90 (median [IQR])	0.5 (0–2.5)	0.4 (0–2.4)	0.5 (0–2.7)	0.5 (0.1–2.6)	0.98
Total sleep time, min (mean ± SD)	422 ± 64	415 ± 72	430 ± 70	408 ± 75	0.20
Sleep efficiency, % (median [IQR])	90.5 (84–95.2)	87.6 (76–92)	84.2 (73–89)	89.6 (79.8–93)	0.07

CAI, central apnea index; HI, hypopnea index; IQR, interquartile range; N(1,2,3), non-rapid eye movement stages of sleep; OAI, obstructive apnea index; ODI, oxygen desaturation index; RDI, respiratory disturbance index; REM, rapid eye movement stage of sleep; SD, standard deviation; T90, proportion of cumulative sleep time with oxygen saturation below 90% in total sleep time.

### 3.1 Correlation between manual and automatic RDI scoring

**Figure 1:**
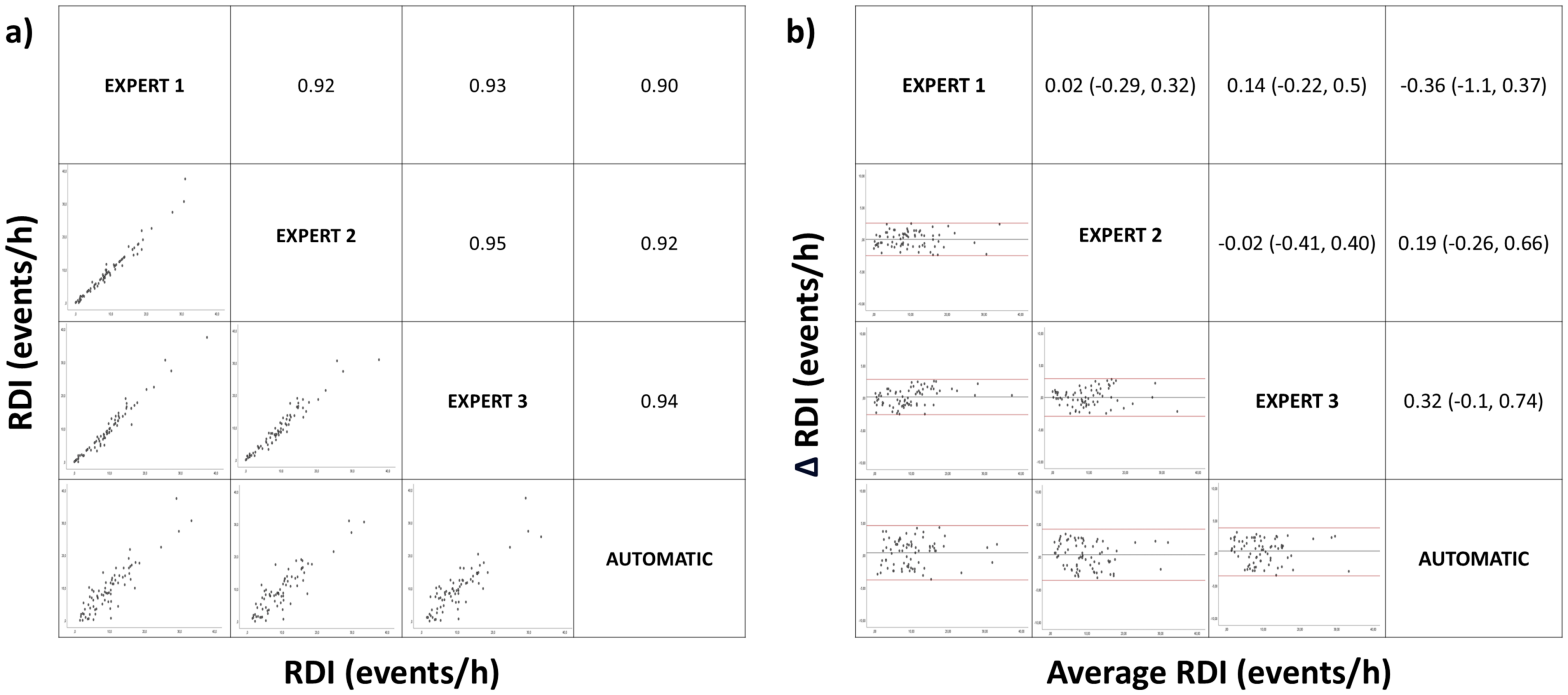
Correlation matrix (left panel) and Bland–Altman (right panel) plots for the respiratory disturbance index (RDI). Pearson correlation coefficients and the average bias (95% confidence interval) are in the left and right panels, respectively.

We found a high correlation in RDI among the three manual scorings and between manual and automatic scoring (Figure 1a). The mean correlation (Pearson correlation coefficient) between the three manually scored RDIs was 0.93 (95% confidence interval [CI] 0.92–0.95), while the mean correlation between manual and automatic RDI scoring was 0.92 (95% CI 0.90–0.94). Therefore, interscorer correlation in the manual results was similar to the correlation between manual and automatic results.

When comparing the mean difference in manual RDI scorings versus the mean difference between manual and automatic scorings using the Bland–Altman matrix, we found no significant difference (Figure 1b). The mean difference in manually scored RDI values among the three experts was 0.04 events/h (95% CI −0.27 to 0.30), while the mean difference in manually versus automatically scored RDIs was 0.05 (95% CI −0.39 to 0.56). This mean difference in RDI had diagnostic relevance in only three patients between the three manually scored and in two patients between manual and automatic scoring and for severity classification in three patients for the mild–moderate category and none for the moderate–severe category.

We also calculated ICCs for all combinations of RDI between manual and automated scoring (Supplementary Table S1). There was a high level of agreement between RDI values recorded by the three experts (ICC: 0.93–0.98), and between manual scoring and automatic scoring (ICC: 0.91–0.99). Agreement was maintained when stratified by OSA severity and according to age (children [from 2 year to 6 years], middle childhood [from 6 to 12 years] and teens [from 12 to 16 years]).

In addition, there was substantial agreement between the three experts for OAI and CAI (ICC: 0.60–0.86 and 0.75–0.98, respectively), as well as between manual and automatic scoring (ICC: 0.67–0.86 and 0.72–0.89, respectively). However, there was lower correlation for the HI (ICC: 0.27–0.75 and 0.36–0.68, respectively). Agreement was maintained when stratified by OSA severity and according to age.

Also, there was also adequate agreement between the three experts for ODI (ICC: 0.94–1.00), arousal index (ICC: 0.68–0.94) and T90 (ICC: 0.89–0.99), as well as between manual and automatic scoring (ICC: 0.92–0.99, ICC: 0.69–0.92 and ICC: 0.90–1.00, respectively).

### 3.2 Correlation between manual and automatic scoring of sleep architecture

ANOVA showed no significant differences between the mean percentage and duration of the different sleep phases obtained through manual and automatic scoring (Table 2).

**Figure 2:**
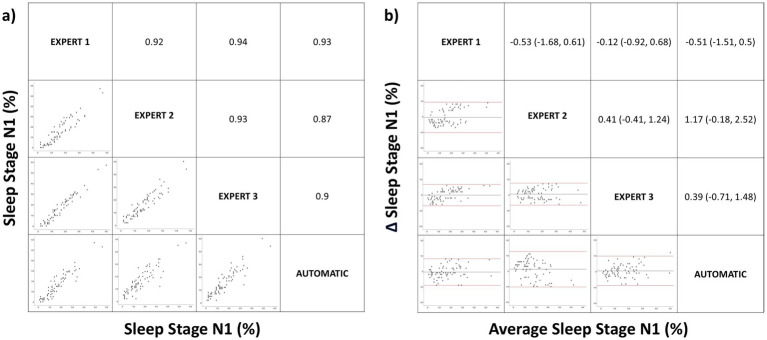
Correlation matrix (left panel) and Bland–Altman (right panel) plots for the sleep stage N1 (%). Pearson correlation coefficients and the average bias (95% confidence interval) are in the left and right panels, respectively.

**Figure 3:**
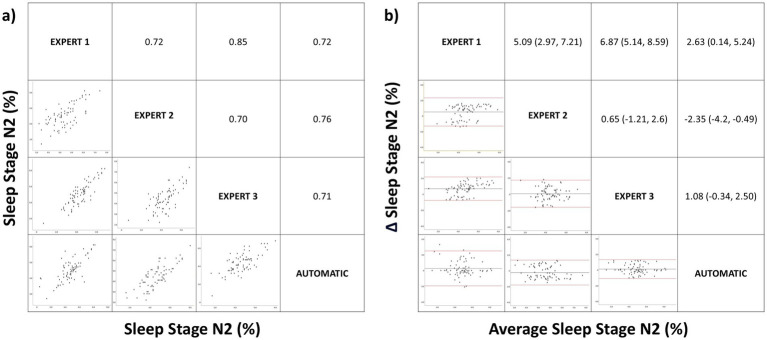
Correlation matrix (left panel) and Bland–Altman (right panel) plots for the sleep stage N2 (%). Pearson correlation coefficients and the average bias (95% confidence interval) are in the left and right panels, respectively.

**Figure 4:**
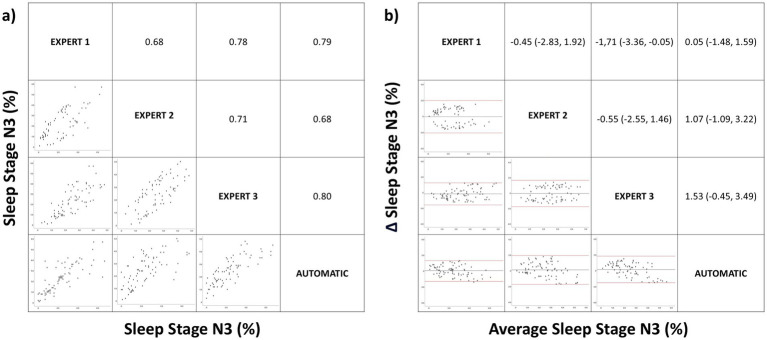
Correlation matrix (left panel) and Bland–Altman (right panel) plots for the sleep stage N3 (%). Pearson correlation coefficients and the average bias (95% confidence interval) are in the left and right panels, respectively.

**Figure 5:**
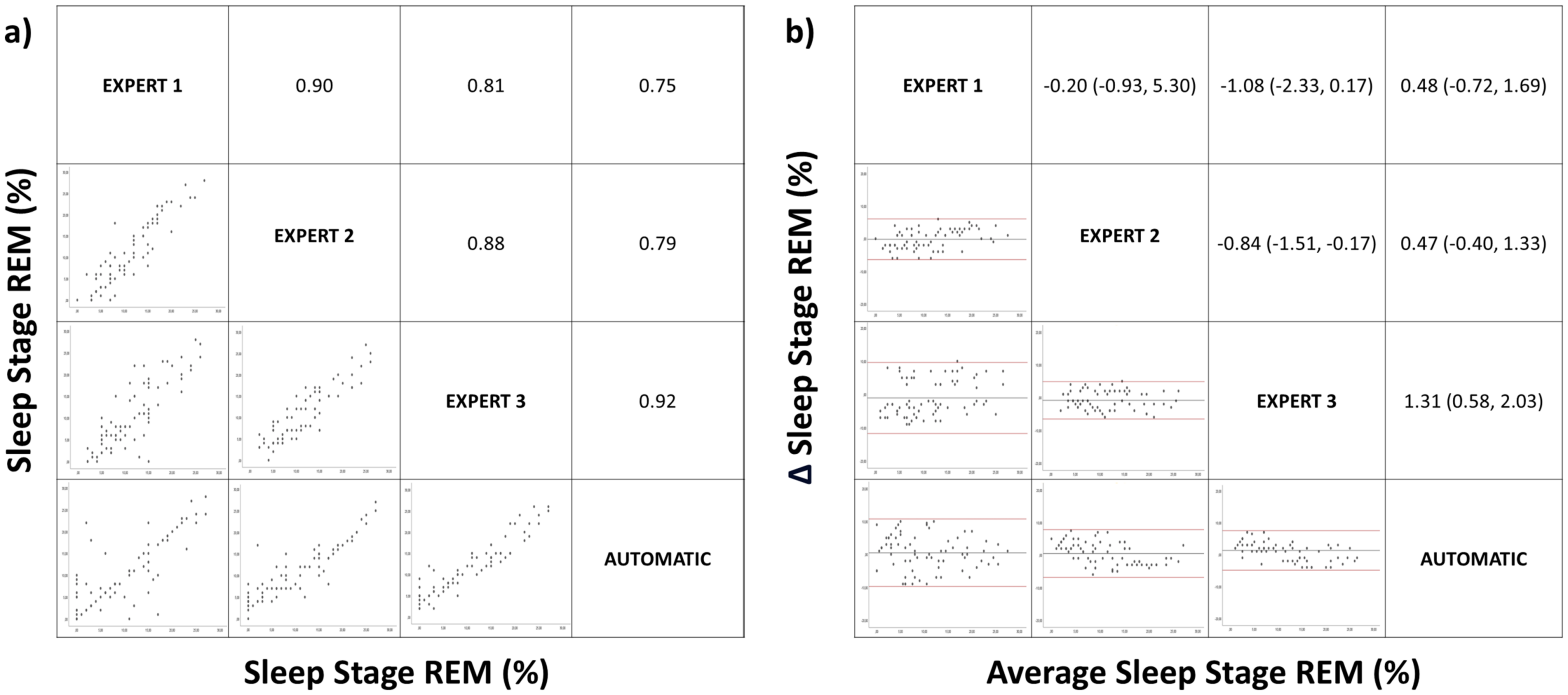
Correlation matrix (left panel) and Bland–Altman (right panel) plots for the sleep stage rapid eye movement (REM). Pearson correlation coefficients and the average bias (95% confidence interval) are in the left and right panels, respectively.

Figures 2–5 show the results of the bivariate and Bland–Altman analysis of the different sleep architecture variables, comparing the different manual scorings and the manual and automated scorings. For N1(%), the mean correlation between manual scorings was 0.93, while the mean correlation between manual and automatic scoring was 0.90. The Bland–Altman analysis (Figure 2b) showed that the mean differences between manual scorings and between manual and automatic scoring were not significantly different (−0.08% vs. 0.35%, *p* > 0.05).

For N2(%), the mean correlation of manually scored results was 0.76. When we compared the mean correlation between the different manual scorings versus the mean correlation between manual and automatic scoring, we found no significant difference (0.76 vs. 0.73, *p* > 0.05). The Bland–Altman analysis of stage N2 (Figure 3b) showed that the mean difference between automated and manual scoring was significantly lower than the mean difference between manual scorings (0.45% vs. 4.2%, *p* = 0.01).

Similarly, for N3(%), there was good correlation between the different manual scorings and between manual and automatic scoring (Figure 4a). When we compared the mean differences between the different manual scorings versus between manual and automated scoring using Bland–Altman plots for N3 (Figure 4b), we found no significant difference (−0.90% vs. 0.88%, *p* > 0.05).

For the REM stage, there was adequate correlation between manual and automatic scoring without significant differences between the different manual scorings or between manual and automatic scoring (Spearman correlation coefficient 0.86 vs. 0.82, *p* > 0.05), as shown in Figure 5a. The Bland–Altman analysis (Figure 5b), which compared the mean differences between the three manual scorings versus between manual and automated scoring, showed no significant difference (−0.71% vs. 0.75%, *p* > 0.05). The correlation observed in sleep architecture was maintained in total sleep time (Pearson correlation coefficient between different manual scorings versus between manual and automatic scoring: 0.84 vs. 0.78, *p* > 0.05). The correlation was lower for sleep efficiency, without a significant difference (Spearman coefficient: 0.51 vs. 0.44, *p* > 0.05).

We also calculated ICCs between manual and automated scoring for all combinations of sleep stages (Supplementary Table S2). There was adequate agreement between the three experts for all sleep phases (ICC of N1[%]: 0.91–0.98; N2[%]: 0.65–0.94; N3[%]: 0.72–0.91 and ICC of REM[%]:0.85–0.97), as well as between manual and automatic scoring (ICC of N1[%]: 0.88–0.98; N2[%]: 0.78–0.92; N3[%]: 0.66–0.91 and ICC of REM[%]:0.81–0.97). The agreement was maintained when the data were stratified by OSA severity. However, when the data were stratified by age, there was a lower correlation between the manual and automatic corrections for (N1%) and N2(%) in middle childhood (from 6 to 12 years), and particularly in children (from 2 year to 6 years).

## 4 Discussion

Our study is the first validation study of Somnolyzer 24×7 computer-assisted sleep scoring system in pediatric population. Our results evidenced that automatic polysomnography scoring system based on AI showed strong correlation with manual scoring in respiratory events and sleep architecture.

Automatic scoring systems have been used in sleep studies for more than 20 years. The first algorithms were able to detect desaturations, snoring, heart rate, and position (30, 31), whereas current algorithms can also detect sleep stages, respiratory events, and arousals using one or more channels, evaluating the spectral power threshold of the frequency bands and detecting waveforms through pattern recognition (32, 33). Although several automatic sleep study correction systems have been validated with high correlations to manual corrections, only Somnolyzer (Philips Respironics), Ensosleep (Ensodata) and Domino (Somnomedics AG) have been certified by the AASM. Regarding Somnolyzer, the latest version includes an AI classifier with bi-directional long-short-term RNN which allows a more accurate correction. Several studies in adults evidenced a high correlation in both sleep architecture and the detection of respiratory events (20–28). Despite the potential benefits of automatic scoring systems, validation of Somnolyzer 24×7 in the pediatric population has not yet been realized.

Polysomnography scoring is laborious and technically complex, with differences in the definitions of respiratory events in children versus adults. Despite the potential benefits of automatic scoring systems, Somnolyzer 24×7 has not yet been validated in the pediatric population. Automated scoring systems with proven validity and reproducibility could increase access to pediatric polysomnography in sleep units. Our validation study of the Somnolyzer 24×7 automatic scoring system showed a high level of agreement between manual and automatic scoring in sleep architecture and respiratory events. Manual scoring served as our reference standard, though it is limited by the inherent interscorer variability. To mitigate this limitation, we compared the agreement between the different manual scorings versus the agreement between manual and automatic scoring.

Regard the scoring of respiratory events, we found no significant differences when comparing the means using ANOVA for manual versus automatic correction for respiratory events and RDI. Furthermore, the analysis of the degree of agreement using the ICC revealed a high agreement in RDI, a substantial agreement in OAI and CAI, and a moderate agreement in HI. HI maintains a strong positive linear correlation between manual and automatic correction. This lower agreement in HI may be due to a detection of obstructive apneas rather than hypopneas and has no relevance to RDI. In addition, there was a strong positive linear relationship between manual and automatic scoring (Figure 1a) in RDI with a low mean difference detection which has no diagnostic relevance (Figure 1b). These results demonstrate that Somnolyzer 24×7 shows a high level of correlation with expert correction in the detection of respiratory events. Furthermore, this high level of correlation is maintained when the data is stratified by age group and OSA severity.

Concerning to sleep architecture, we found no significant differences when comparing the means using ANOVA for manual versus automatic correction for sleep stages. However, there is a tendency toward statistical significance in N2. In the analysis of the degree of agreement using the ICC there was an almost perfect agreement in all sleep stages but when the data were stratified by age, there was a lower correlation between the manual and automatic corrections for (N1%) and N2(%) in middle childhood, and particularly in children. This may be because the Somnolyzer 24×7 has only been validated in adults, there are fewer studies of pediatric patients, and sleep wave differences exist between adults and pediatric patients. During initial years of life electroencephalogram power increases in particular in the slow wave frequency range during NREM sleep. Then, maximal power values in the faster frequency bands, such as theta, alpha and beta activity, are reached at 2–5 years of age. Power in these frequency bands decreases steadily thereafter (34). This difference in the sleep waveform, especially in children, justifies the discrepancy between manual and automatic correction and also the results with a tendency toward statistical significance in the mean comparison analysis on sleep efficiency. Regarding the other results, there was a very strong positive linear relationship between manual and automatic scoring in N1(%) (Figure 2a), a strong positive linear relationship in N2(%), N3(%) and REM(%) (Figures 2a–5a) with a low mean difference detection in all sleep stages which has no diagnostic relevance (Figures 2b–5b). Therefore, automatic correction shows a very high degree of correlation with manual correction with respect to sleep architecture, despite limitations arising from changes in sleep waveforms in the pediatric population relative to adults.

In a validation study of the Somnolyzer system in adults (20), Punjabi and colleagues found a very strong correlation between manual and automatic scoring of AHI (Pearson correlation coefficient: 0.93), practically the same value that we obtained for RDI (Pearson correlation coefficient: 0.92). For sleep architecture, we found a stronger correlation in most sleep phases compared with Punjabi and colleagues (N1: 0.90 vs. 0.63, N2: 0.73 vs. 0.66, N3: 0.76 vs. 0.65 and REM: 0.82 vs. 0.92). This difference may be attributable to the different versions of Somnolyzer employed. While we used Somnolyzer 4.0, which classifies respiratory events and sleep architecture using artificial intelligence (RNN), Punjabi and colleagues used version 3.0. The main changes of Somnolyzer 4.0 are spindle detection from both hemispheres, improved slow-wave detection using empirical mode decomposition (improve rule-based sub-classification of NREM sleep stages), new channels and the use of LSTM and RNN classifiers with a confidence score based on RNN output probabilities.

The results obtained in our study promote the implementation of polysomnography in sleep units that do not currently perform it on pediatric patients with suspected OSA and may reduce waiting lists in units that do perform polysomnography in pediatric patients. The Somnolyzer 24×7 software is user-friendly and uses traffic light color coding to indicate the confidence of its scoring. This allows sleep reviewers to focus on the yellow and red codes, improving time efficiency. However, the implementation of automated sleep study correction systems requires caution to avoid over-reliance on AI and requires clinician training, so we propose a hybrid scoring workflow, using Somnolyzer 24×7 as a complementary to enhance and standardized sleep staging and improve patient diagnosis.

Despite our results of Somnolyzer 24×7 validation in patients with suspected OSA, further validation is required in pediatric patients with comorbidities, patients at different stages of child development and patients with sleep disorders other than OSA, such as insomnia, circadian rhythm disturbances, parasomnias, narcolepsy, idiopathic or recurrent hypersomnia, restless legs syndrome, and periodic leg movement disorder.

One main limitation of our study is that the three sleep medicine experts worked in the same clinic so we recommend the development of multicenter external validation studies. Another limitation is that we did not perform both manual and automatic joint correction. Therefore, randomized trials in pediatric population are needed to compare correction by AI alone, manual correction alone, and combined correction by AI and manual correction. A further limitation is the heterogeneity of the population, as we included children from all stages of development. However, when we analyzed the data by age group, we found that there were high correlations between respiratory events and sleep stages across the age groups, except for the correlation between manual and automatic corrections for N1% and N2%.

The main strengths of our study are its prospective and robust methodology, which has been replicated in other validation studies, and the involvement of three medical experts in manual scoring, allowing for a more reliable comparison with automated scoring. Another strength is the sample size and the favorable results obtained despite the technical difficulties of performing polysomnography in pediatric patients.

## 5 Conclusion

Somnolyzer 24×7 automatic polysomnography scoring system showed strong correlation with manual scoring in respiratory events and sleep architecture. By simplifying the diagnostic process, this automatic system may facilitate broader implementation of polysomnography in sleep units.

## Data Availability

The original contributions presented in the study are included in the article/Supplementary material, further inquiries can be directed to the corresponding author.
