# Supplementary figures and images for: Validation of the Somnolyzer 24×7 automatic scoring system in children with suspected obstructive sleep apnea
Source: Front Med (Lausanne). 2025 Jun 18;12:1617530. doi: 10.3389/fmed.2025.1617530 (PMC12213436; doi:10.3389/fmed.2025.1617530)

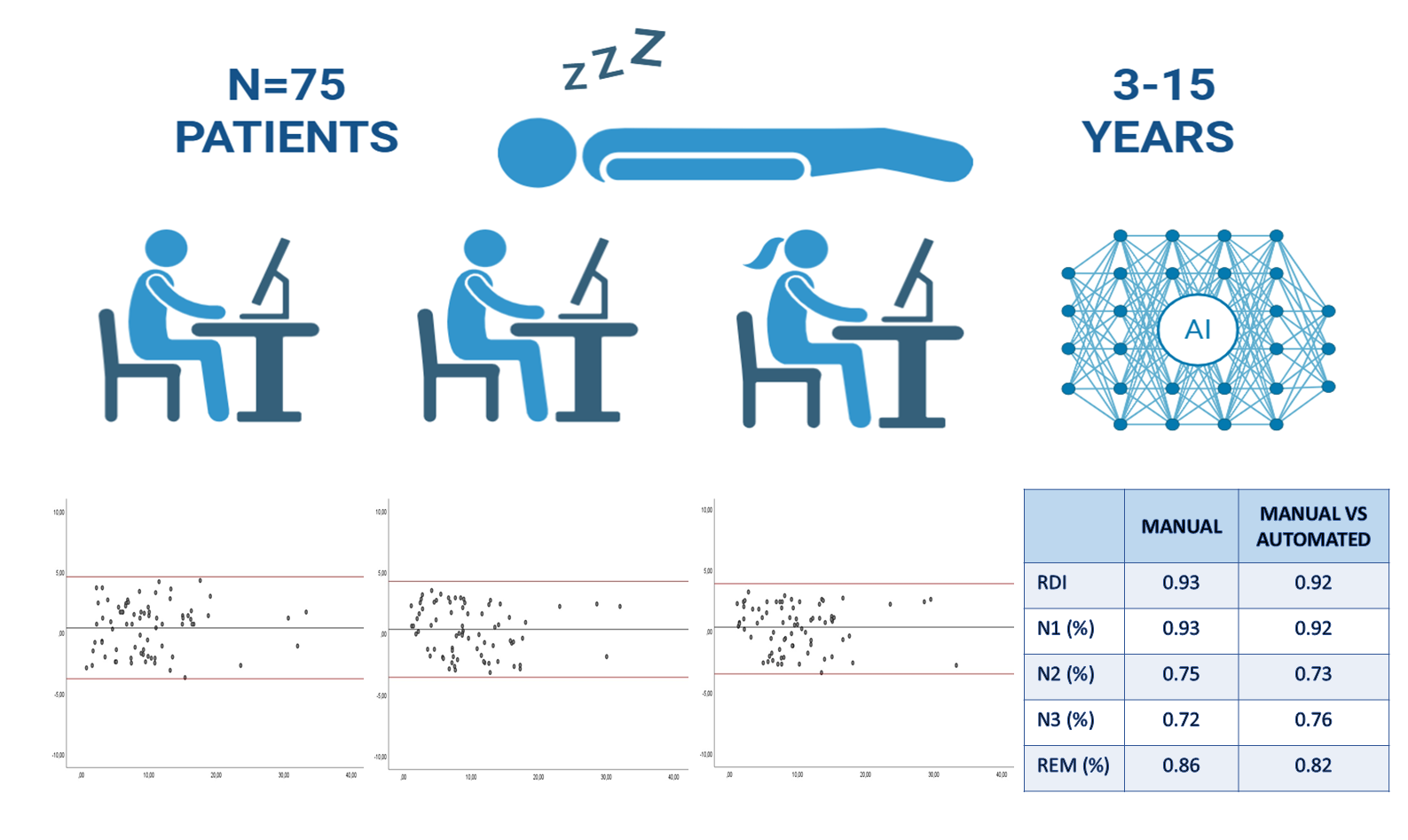

Supplement: Supplementary file 1 [file Image_1.tif]
