# Supplementary material for: Validation of the Somnolyzer 24×7 automatic scoring system in children with suspected obstructive sleep apnea
Source: Front Med (Lausanne). 2025 Jun 18;12:1617530. doi: 10.3389/fmed.2025.1617530 (PMC12213436; doi:10.3389/fmed.2025.1617530)
Supplement: Supplementary file 2 [file Table_1.docx]

Supplementary Material

# Supplementary Figures and Tables

## Supplementary Tables

**Table S1**: Intraclass correlation coefficient with 95% confidence interval of respiratory events, oxygen desaturation index, and proportion of time with oxygen saturation below 90%.

|  | EXPERT 1 | EXPERT 2 | EXPERT 3 |
| --- | --- | --- | --- |
| **RDI** |  |  |  |
| EXPERT 1 | — | — | — |
| EXPERT 2 | 0.96 (0.93 to 0.97) | — | — |
| EXPERT 3 | 0.96 (0.94 to 0.98) | 0.97 (0.96 to 0.98) | — |
| SOMNOLYZER | 0.94 (0.91 to 0.96) | 0.98 (0.97 to 0.99) | 0.98 (0.97 to 0.99) |
| **OAI** |  |  |  |
| EXPERT 1 | — | — | — |
| EXPERT 2 | 0.79 (0.71 to 0.86) | — | — |
| EXPERT 3 | 0.69 (0.65 to 0.76) | 0.64 (0.60 to 0.69) | — |
| SOMNOLYZER | 0.74 (0.67 to 0.82) | 0.76 (0.7 to 0.84) | 0.77 (0.68 to 0.86) |
| **CAI** |  |  |  |
| EXPERT 1 | — | — | — |
| EXPERT 2 | 0.79 (0.75 to 0.85) | — | — |
| EXPERT 3 | 0.94 (0.87 to 0.98) | 0.80 (0.77 to 0.84) | — |
| SOMNOLYZER | 0.81 (0.75 to 0.85) | 0.78 (0.72 to 0.85) | 0.82 (0.76 to 0.89) |
| **HYPOPNEA INDEX** |  |  |  |
| EXPERT 1 | — | — | — |
| EXPERT 2 | 0.68 (0.64 to 0.75) | — | — |
| EXPERT 3 | 0.37 (0.31 to 0.48) | 0.29 (0.27 to 0.38) | — |
| SOMNOLYZER | 0.49 (0.46 to 0.58) | 0.43 (0.36 to 0.5) | 0.57 (0.52 to 0.68) |
| **ODI** |  |  |  |
| EXPERT 1 | — | — | — |
| EXPERT 2 | 0.98 (0.95 to 0.99) | — | — |
| EXPERT 3 | 0.97 (0.94 to 0.99) | 0.99 (0.98 to 1.00) | — |
| SOMNOLYZER | 0.96 (0.92 to 0.99) | 0.97 (0.94 to 0.99) | 0.98 (0.96 to 0.99) |
| **AROUSAL INDEX** |  |  |  |
| EXPERT 1 | — | — | — |
| EXPERT 2 | 0.82 (0.76 to 0.84) | — | — |
| EXPERT 3 | 0.74 (0.68 to 0.79) | 0.89 (0.82 to 0.94) | — |
| SOMNOLYZER | 0.87 (0.82 to 0.92) | 0.83 (0.79 to 0.87) | 0.74 (0.69 to 0.78 |
| **T90** |  |  |  |
| EXPERT 1 | — | — | — |
| EXPERT 2 | 0.94 (0.92 to 0.98) | — | — |
| EXPERT 3 | 0.96 (0.94 to 0.99) | 0.93 (0.89 to 0.96) | — |
| SOMNOLYZER | 0.95 (0.92 to 0.97) | 0.94 (0.90 to 0.98) | 0.98 (0.96 to 1.00) |

**Abbreviations:** CAI, central apnea index; OAI, obstructive apnea index; ODI, oxygen desaturation index; RDI, respiratory disturbance index; T90, proportion of cumulative sleep time with oxygen saturation below 90% in total sleep time.

Table S2: Intraclass correlation coefficient with the 95% confidence interval of the different sleep phases.

|  | EXPERT 1 | EXPERT 2 | EXPERT 3 |
| --- | --- | --- | --- |
| **N1 (%)** |  |  |  |
| EXPERT 1 | — | — | — |
| EXPERT 2 | 0.95 (0.91 to 0.97) | — | — |
| EXPERT 3 | 0.97 (0.96 to 0.98) | 0.96 (0.94 to 0.98) | — |
| SOMNOLYZER | 0.96 (0.94 to 0.98) | 0.93 (0.88 to 0.95) | 0.94 (0.91 to 0.96) |
| **N2 (%)** |  |  |  |
| EXPERT 1 | — | — | — |
| EXPERT 2 | 0.78 (0.65 to 0.86) | — | — |
| EXPERT 3 | 0.90 (0.85 to 0.94) | 0.82 (0.71 to 0.89) | — |
| SOMNOLYZER | 0.87 (0.80 to 0.92) | 0.86 (0.78 to 0.91) | 0.83 (0.72 to 0.89) |
| **N3 (%)** |  |  |  |
| EXPERT 1 | — | — | — |
| EXPERT 2 | 0.85 (0.76 to 0.90) | — | — |
| EXPERT 3 | 0.86 (0.78 to 0.91) | 0.82 (0.72 to 0.89) | — |
| SOMNOLYZER | 0.86 (0.78 to 0.91) | 0.78 (0.66 to 0.86) | 0.83 (0.74 to 0.90) |
| **REM (%)** |  |  |  |
| EXPERT 1 | — | — | — |
| EXPERT 2 | 0.95 (0.91 to 0.97) | — | — |
| EXPERT 3 | 0.90 (0.85 to 0.94) | 0.94 (0.91 to 0.96) | — |
| SOMNOLYZER | 0.88 (0.81 to 0.93) | 0.93 (0.89 to 0.95) | 0.95 (0.92 to 0.97) |

**Abbreviations:** N(1,2,3), non-rapid eye movement stages of sleep; REM, rapid eye movement stage of sleep
